# ILC3, a Central Innate Immune Component of the Gut-Brain Axis in Multiple Sclerosis

**DOI:** 10.3389/fimmu.2021.657622

**Published:** 2021-04-12

**Authors:** Đorđe Miljković, Bojan Jevtić, Ivana Stojanović, Mirjana Dimitrijević

**Affiliations:** Department of Immunology, Institute for Biological Research “Siniša Stanković” - National Institute of Republic of Serbia, University of Belgrade, Belgrade, Serbia

**Keywords:** ILC3 cells, multiple sclerosis, gut-associated lymphoid tissues (GALT), Treg - regulatory T cell, Th17 (T helper 17 cell), AhR (Aryl hydrocarbon Receptor), FFAR2 (GPR43), TLR2

## Abstract

Gut immune cells have been increasingly appreciated as important players in the central nervous system (CNS) autoimmunity in animal models of multiple sclerosis (MS). Among the gut immune cells, innate lymphoid cell type 3 (ILC3) is of special interest in MS research, as they represent the innate cell counterpart of the major pathogenic cell population in MS, *i.e.* T helper (Th)17 cells. Importantly, these cells have been shown to stimulate regulatory T cells (Treg) and to counteract pathogenic Th17 cells in animal models of autoimmune diseases. Besides, they are also well known for their ability to stabilize the intestinal barrier and to shape the immune response to the gut microbiota. Thus, proper maintenance of the intestinal barrier and the establishment of the regulatory milieu in the gut performed by ILC3 may prevent activation of CNS antigen-specific Th17 cells by the molecular mimicry. Recent findings on the role of ILC3 in the gut-CNS axis and their relevance for MS pathogenesis will be discussed in this paper. Possibilities of ILC3 functional modulation for the benefit of MS patients will be addressed, as well.

## Introduction

One of the major open questions about multiple sclerosis (MS) pathogenesis is how the autoimmune response directed against the central nervous system (CNS) is initiated. It is not only that we have not been able to identify preliminary antigens that the autoimmunity is directed against, but also the place of the initial activation of the autoimmune response remains elusive. Gut microbiota has been increasingly studied as the source of antigens that can activate CNS-specific autoreactive T cells, while gut-associated lymphoid tissues (GALT) have been considered as the potential site of their initial activation. MS pathogenesis essentials are presented in **Box 1**, while details can be found in numerous review papers (1–4). In the following chapters, we will present current knowledge on the role of gut microbiota and GALT in the etiopathogenesis of MS, with an emphasis on the role of intestinal innate lymphoid cells type 3 (ILC3) in the process.

Box 1 **Multiple sclerosis (MS) essentials**

- MS is chronic inflammatory, demyelinating and neurodegenerative disease of the central nervous system (CNS).Typical neurological symptoms of MS comprise diminished sensory and visual perception, motor dysfunction, fatigue, pain, and occasionally cognitive deficit. Most MS patients exhibit a relapsing-remitting course of the disease, distinguished by alternations between acute attacks and remission phases. Also, MS may present clinically isolated syndrome or progressive (primary and secondary) clinical course.
- Autoimmune response against the CNS resulting in the CNS inflammatory infiltrates has a major contribution to MS pathogenesis.IFN-γ-producing Th1 cells and IL-17-producing Th17 cells, defined by the expression of T-bet and RORγt master regulators, respectively, enter the brain at semipermeable and damaged sites of the BBB and initiate neuroinflammation. Neuroinflammation induces the opening of BBB and enables the second wave of immune cell entry into the CNS and the formation of brain lesions. CD8^+^ T cells, B cells, and macrophages (Mf) have the leading role in the CNS tissue destruction CD4^+^ T regulatory cells (Treg), defined by the expression of CD25 and Foxp3 as a master transcription factor, operate at the opposite arm of neuroinflammation to reduce/recover damage.
- The etiology of MS is multifactorial and involves interaction between intrinsic (genetic) and extrinsic (environmental) risk factors that influence either innate or adaptive immunity. Despite the conclusive autoimmune trait of MS, the precise trigger for the CNS-directed autoimmune response is still unknown.Autoreactive T cells in the blood of MS patients display specificity for multiple myelin protein-derived antigens such as myelin basic protein (MBP), proteolipid protein, and myelin oligodendrocyte glycoprotein (MOG). However, none of these myelin protein-derived antigens is recognized as a dominant antigen in MS, while T cells of the same specificity exist in the blood of healthy individuals. Experimental autoimmune encephalomyelitis (EAE) is an animal model of MS that is induced in susceptible animals through immunization with the CNS antigens.

## The Gut Immune System in the Intricacy of the CNS Autoimmunity

Apart from the induction of immune responses against harmful microorganisms and maintaining immune homeostasis in the gut, the immune system of the gut intercedes between intestinal microbiota/metabolites and autoimmune responses. Immune cells are highly enriched in the GALT organized in the forms of Peyer’s patches, isolated lymphoid follicles, and scattered among the intestinal epithelial cells and in the lamina propria across the gastrointestinal tract. Also, the immune system of the gut encompasses gut-draining lymph nodes that have intensive communication with the GALT. Recently disclosed changes in immune cells composition and accumulation within different GALT compartments in EAE animals (5–13) support the concept that initiation and/or regulation of autoimmune response to CNS antigens may occur in the gut.

The gut microenvironment participates in the shaping of autoimmune responses to CNS antigens presumably by modulating the activation/differentiation of autoreactive T cells and guiding their trafficking to the CNS. Potentially encephalitogenic T cells were shown to migrate into the gut, where they were further activated towards pathogenic population, or they were modulated to become regulatory cells (7, 14, 15). Accordingly, enhanced Th17 induction in response to segmented filamentous bacteria was described in the small intestine of mice, in particular in the terminal ileum (16–18), while excessive Th17 expansion in the small intestine of humans was associated with MS activity (19). Also increased numbers of Th1/Th17 cells and decreased numbers of Treg cells were found in the gut lamina propria, Peyer’s patches, and mesenteric lymph nodes of mice with experimental autoimmune encephalomyelitis (EAE) before the appearance of clinical symptoms, as well as at the disease peak (9). Increased intestinal permeability, alterations in tight junction functioning, and modifications in intestinal morphology occurred along with the changes in the T cells composition in GALT, thus indicating that disruption of intestinal homeostasis was dependent on the immune response at the initiation of EAE (9). Even more, it has been suggested that the very initiation of MS may occur in the GALT through the process of molecular mimicry and/or as a consequence of the loss of gut barrier integrity (20–22).

**Figure 1:**
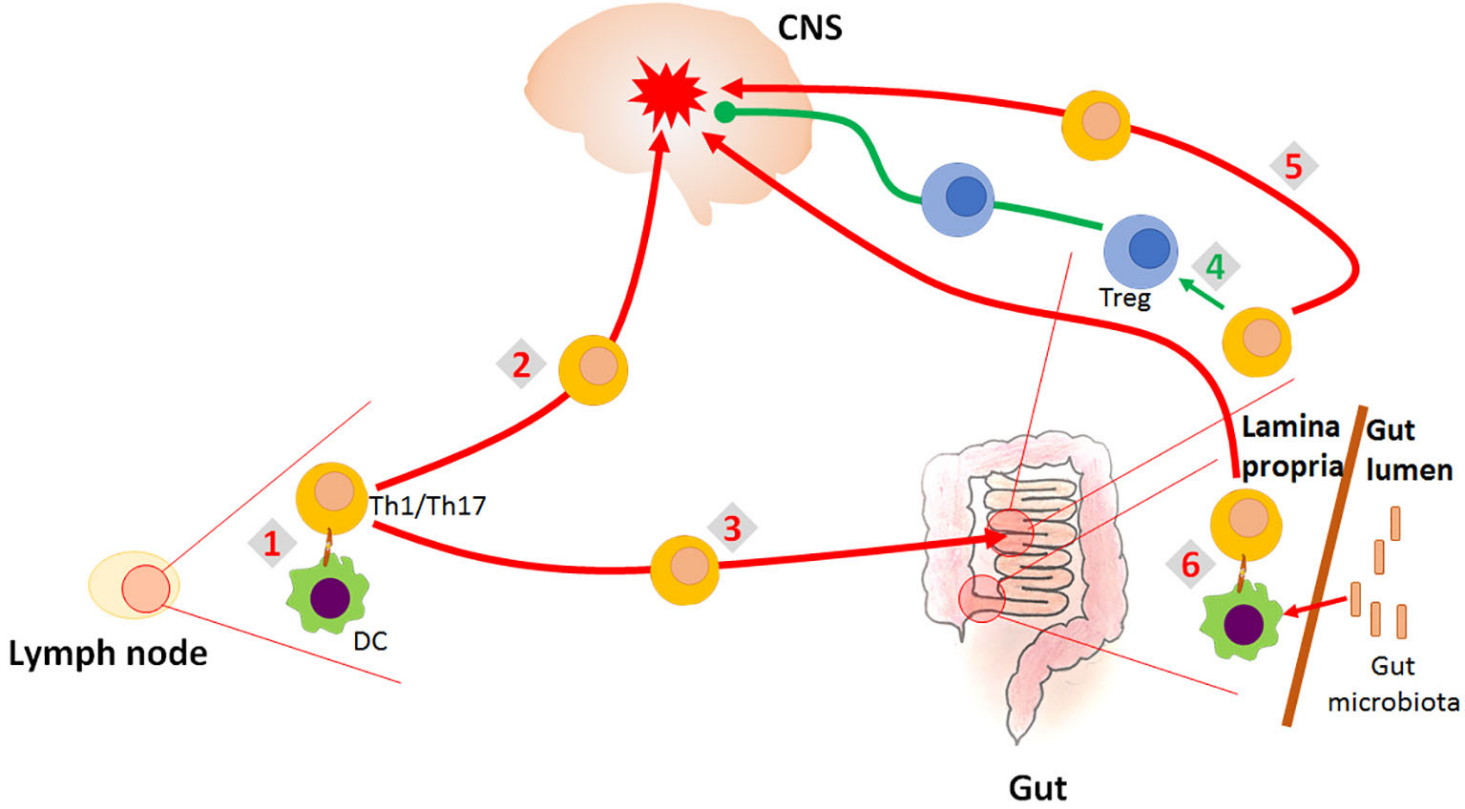
Role of the gut in the CNS autoimmunity. CNS-autoreactive Th1 and Th17 cells are activated in the lymph nodes (1). They migrate into the CNS where they initiate inflammatory response imposing destruction of the CNS tissue (2). They also migrate into the gut (3), where they can be re-differentiated to Treg which counteract the inflammation in the CNS (4). However, they can also be supported by the gut environment in their encephalitogenicity (5). Finally, it is proposed that encephalitogenic Th cells might be initially activated in the gut by the process of molecular mimicry, as they cross-react with gut microbial antigens (6).

Conversely, GALT is involved in establishing tolerance to orally administered (auto)antigens including peptides from the nervous tissue. Increased apoptosis of autoreactive T cells in myelin basic protein (MBP)-fed mice occurs in Peyer’s patches, thus indicating that Peyer’s patches are the principal site for oral tolerance induction in the MBP-specific model of EAE (5). Furthermore, suppression of EAE induced by CD3-specific antibody treatment was presumably reflected by conversion of myelin oligodendrocyte glycoprotein (MOG)-specific Th17 cells into regulatory phenotype occurring in the small intestine (7). It is assumed that autoreactive T cells experiencing phenotypic adaptation in the GALT attain characteristics that favor their migration to the brain (20). Trafficking of CNS-specific autoreactive cells to the gut is mediated through α4β7- MAdCAM-1 (mucosal addressin cell adhesion molecule 1) interaction. Protection from MOG_35−55_-induced EAE in MAdCAM-1-deficient mice was accompanied by impaired migration of MOG_35-55_-activated lymphocytes to small intestine lamina propria and Peyer’s patches (12). Infiltration of colonic lamina propria with MOG-specific Th17 cells, also dependent on α4β7-MAdCAM-1 pathway, in the preclinical phase of EAE, has been demonstrated in both active and adoptive transfer EAE models in mice (13). These findings support the notion that recruitment of encephalitogenic T cells to the GALT occurs before immigration into the CNS. However, data are showing that IL-4, co-expressed in Th17 cells or used for treatment in EAE mice, redirected trafficking of pro-inflammatory Th17 cells from the CNS and draining lymph nodes to the mesenteric lymph nodes and ameliorated the disease (10). This effect was achieved through IL-4 dependent increase of retinoic acid (RA) production in dendritic cells (DC) and further induced expression of gut-homing receptors CCR9 and α4β7 on Th cells. Moreover, retaining the autoreactive pro-inflammatory T cells within the intestine has been associated with the resistance to EAE induction in mice (15). It seems that GALT controls CNS-directed autoimmune responses by providing a microenvironment for the activation and differentiation of both encephalitogenic Th cells and Tregs (that may halt these autoreactive T cells). The relationship between the gut and the CNS autoimmunity is shown in **Figure 1**.

Different subpopulations of immune cells residing in GALT that might contribute to CNS autoimmunity comprise conventional lymphocytes (CD4^+^ Th cells, Tregs, CD8^+^ T cytotoxic cells), antigen-presenting, and phagocytic cells (DC and macrophages - Mf), and non-conventional lymphocytes, i.e., ILC. Recent findings disclosed the crucial role of the TGF-β-Smad7 regulatory pathway in the generation of CNS autoreactive Th cells in the intestine as Smad7 inhibited induction of Treg by TGF-β (23). Furthermore, decreased TGF-β signaling with a shift toward inflammatory T cell subtypes was demonstrated in intestinal biopsies from MS patients (23). However, it is acknowledged that TGF-β in combination with pro-inflammatory cytokines promotes Th17 differentiation. Intestinal DC expressing αvβ8 were shown to convert latent TGF-β to an active form and thus favor the generation of Th17 and IL-17-mediated CNS inflammation (24, 25). Besides, in EAE mice the frequency of DC was inversely correlated with the frequency of CD39^+^ Tregs in GALT (26). Considering that DC in GALT present primarily the target for manipulation of orally induced tolerance, it was shown that in orally-tolerated EAE mice intestinal lamina propria γδ T cells secrete XCL1 to promote migration of tolerogenic DC to mesenteric lymph nodes where they induce Tregs (27). Gut-derived IgA-secreting plasma cells in the CNS were recently shown to limit neuroinflammation *via* the production of IL-10 (28). Conversely accumulation of IgA-producing cells reactive with gut bacterial strains associated with MS correlated with acute inflammation in MS (29).

The recently identified ILC primarily involved in regulating intestinal immune responses have also been implicated in CNS autoimmunity. Among different subpopulations of ILC, ILC3 have raised special attention due to the functional similarities with the Th17 that are the major players in CNS inflammation. Indeed, ILC3 share the signature transcription factor retinoid-related orphan receptor γt (RORγt) with Th17 and produce the same major cytokines as Th17 (30).

## ILC3 as the Central Regulators of the Gut Immunity

Immature ILC develop in the bone marrow from common lymphoid progenitor and they tend to migrate to mucosal tissues, although some populate lymphoid tissues, including the spleen and lymph nodes and non-lymphoid organs, such as liver, brain and pancreas (31–34). Also, differentiated ILC3 were found in the bloodstream during a T-cell mediated autoimmune inflammatory disease such as psoriasis (35). ILC3 diverge into at least two subsets that differ developmentally, phenotypically and functionally. Lymphoid tissue inducer cells (LTi)-like ILC3 are characterized by surface expression of CCR6, while natural cytotoxicity receptor (NCR)^+^ ILC3 express NKp46 in mice (36).

Mature ILC3 develop in the lamina propria of the intestine due to specific differentiation factors (retinoic acid - RA, polyphenols and microbiota) (37). Once ILC3 populate tissues, they usually do not migrate (38), thus they have to be replenished through regular divisions. Gut ILC3 proliferation is stimulated by cytokines, including IL-18, tumor necrosis factor-like cytokine 1A, IL-1β, IL-23 and IL-2 (39, 40), short-chain free fatty acids (SCFA) and vitamins A and D (41, 42). ILC3 are critical for the generation of the organized lymphoid tissue in the intestinal wall during development, and they regulate microbiota content and the integrity of the intestinal barrier (38, 43).

ILC3 are present in different GALT compartments where they closely interact with other immune cells, including Th1 and Th17 cells, as well as with the major regulatory population of T cells – FoxP3^+^ T cells, i.e. Treg (14). It is assumed that the healthy balance between Th17 and Treg in the gut is the major prerequisite for adequate functioning of the adaptive immune system and prevention of autoimmune diseases. The ratio and function of Treg and Th17 in the gut are largely under the influence of gut microbiota and food constituents (44). It has been documented that ILC3 can efficiently control the effector Th1 and Th17 cells and shift T effector/Treg balance to the regulatory side (45–47).

ILC3 can sense cues originating from the food or microbiota as they express numerous receptors, such as retinoic acid receptor (RAR) (48), vitamin D receptor (VDR) (49), aryl hydrocarbon receptor (AhR) (43, 50), and free fatty acid receptors (FFAR) (51). In response to environmental signals, such as vitamins, indoles, SCFA, as well as to cytokines produced by surrounding cells, ILC3 produce several cytokines, including IL-17A/F, IL-22, GM-CSF and IL-2.

The main role of IL-17 produced by ILC3 is to attract neutrophils to the intestinal tissue in response to bacterial and fungal infections (52–54). ILC3-derived IL-17 is also important for the induction of antimicrobial peptides and tight junction proteins (55).

ILC3 react to IL-1β produced by gut microbiota-stimulated antigen-presenting cells (DC/Mf) by secreting IL-2 which potentiates Treg activity (47), and GM-CSF which stimulates the release of IL-10 and RA from DC/Mf (56). IL-10 and RA also stimulate Treg activity. Of specific interest for the homeostasis in the gut are IL-2-producing ILC3 (47), as they are essential for IL-2-mediated Treg cell maintenance and, consequently, for oral tolerance to dietary antigen in the small intestine. Further, OX40L-expressing ILC3 were shown extremely important for Treg homeostasis in the intestine (57). Also, ILC3 drive the differentiation of T cells towards Treg as they present antigens within MHC class II molecules to T cells, but without co-stimulatory signals (45). Further, gut ILC3 present antigens to effector Th17/Th1 cells, yet without adequate co-stimulation (58), thus causing their inactivation. Even with OX40L expression, MHC class II^+^ ILC3 were shown to regulate effector T cells in acute colitis (59). Thus, ILC3 act in two ways: directly on effector Th17/Th1 cells or through potentiation of Treg that suppress the effector cell activity.

**Figure 2:**
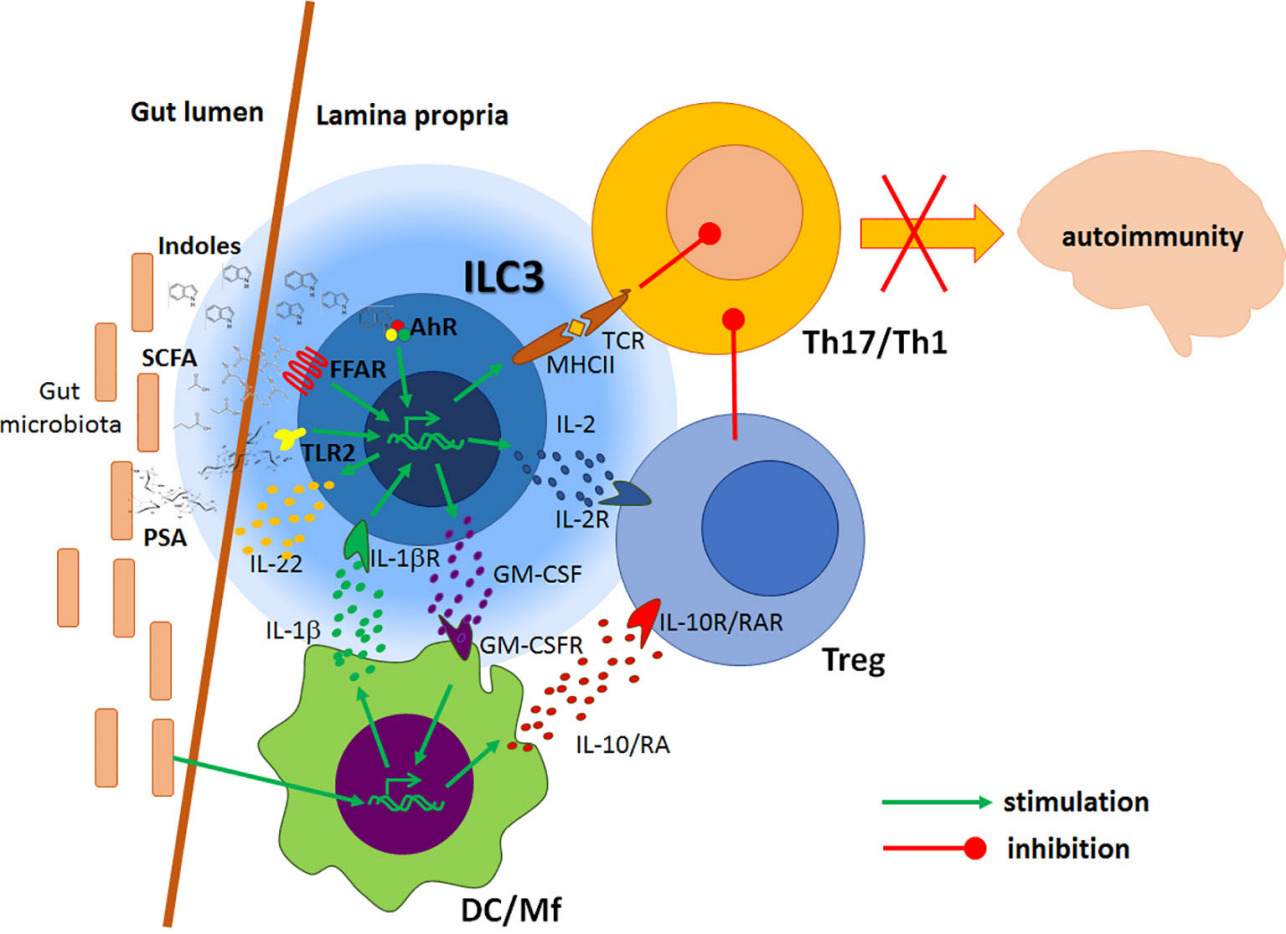
Regulatory effects of gut ILC3 on the CNS autoimmunity. Local antigen-presenting cells (DC/Mf) produce IL-1β under the influence of gut microbiota. IL-1β stimulates ILC3 to produce GM-CSF, which potentiates tolerogenic properties of DC, IL-22 that stimulates intestinal barrier, and IL-2 that favours Treg. DC/Mf also produce IL-10 and retinoic acid (RA) which stimulate Treg activity. ILC3 present antigens to effector Th1 and Th17 cells, but without adequate co-stimulation, thus inhibiting their functions. Products of gut microbiota, such as polysaccharide A (PSA) and processed nutrients, such as short-chain free fatty acids (SCFA) and indoles act on ILC3 through their respective receptors to potentiate their immunomodulatory actions. Consequently, encephalitogenic Th cells are inhibited in the gut, thus ILC3 activity presumably leads to the amelioration of the CNS autoimmunity.

ILC3 are an important source of IL-22, the key cytokine for the stabilization of the intestinal barrier (57). IL-22 keeps intestinal barrier integrity through stimulation of gut epithelial cell turnover, induction of tight junction proteins production, as well as by stimulation of anti-bacterial peptides and mucins generation (60–63). IL-22 and lymphotoxin α produced by ILC3 have the dominant role in epithelial fucosylation involved in the formation of an environmental niche for small intestine commensal bacteria (64). Production of IL-22 by ILC3 is stimulated by multiple biomolecules. IL-1β, IL-18 and IL-23 secreted by DC/Mf stimulate IL-22 production in ILC3 (39, 65–68). ILC3 can recognize lipid antigens through CD1d and consequently generate IL-22 (69). IL-22 production in ILC3 was also shown to be stimulated by a glial-derived neurotrophic factor produced in enteric glial cells in response to TLR ligands (70). Vitamins A and D are potent inducers of IL-22 production by ILC3 (48, 49), as well as AhR ligands and SCFA that act through AhR and FFAR, respectively (50, 71, 72). **Figure 2** illustrates the immunoregulatory activity of gut ILC3 related to CNS autoimmunity.

It has recently been convincingly demonstrated that ILC3 are responsive to circadian regulation (73–75). Importantly, the diurnal rhythm was found affected in EAE (76), while the loss of molecular clock in myeloid cells was found associated with exacerbation of EAE (77). Also, it was reported that IL-22 production in ILC3 and consequent regulation of intestinal barrier function were under the control of vasoactive intestinal peptide (VIP) released from the local enteric neurons (78, 79). VIP release is induced by food consumption, while the functionality of the barrier was inversely correlated with increased growth of epithelial-associated segmented filamentous bacteria. Thus, it is tempting to speculate that disbalanced regulation of the molecular clock in ILC3 contributes to EAE pathogenesis.

Still, it has to be noted that several studies imply pro-inflammatory and disease-promoting activity of ILC3. For example, GM-CSF production by ILC3 was associated with enhanced maturation and polarization of inflammatory intestinal Mf and with the intestinal inflammatory response as observed in colitis (80, 81). Also, MHC class II^+^ ILC3 were shown to co-stimulate effector T cells in chronic colitis (59). The high salt diet was shown to potentiate IL-17 production in ILC3 and subsequent intestinal inflammation (82). Further, as a part of the gut immune response to segmented filamentous bacteria, ILC3 stimulated epithelial serum amyloid A protein production, which in turn promoted Th17 cells (83).

## Untangling Potency of Gut ILC3 Modulation for MS Therapy

**Figure 3:**
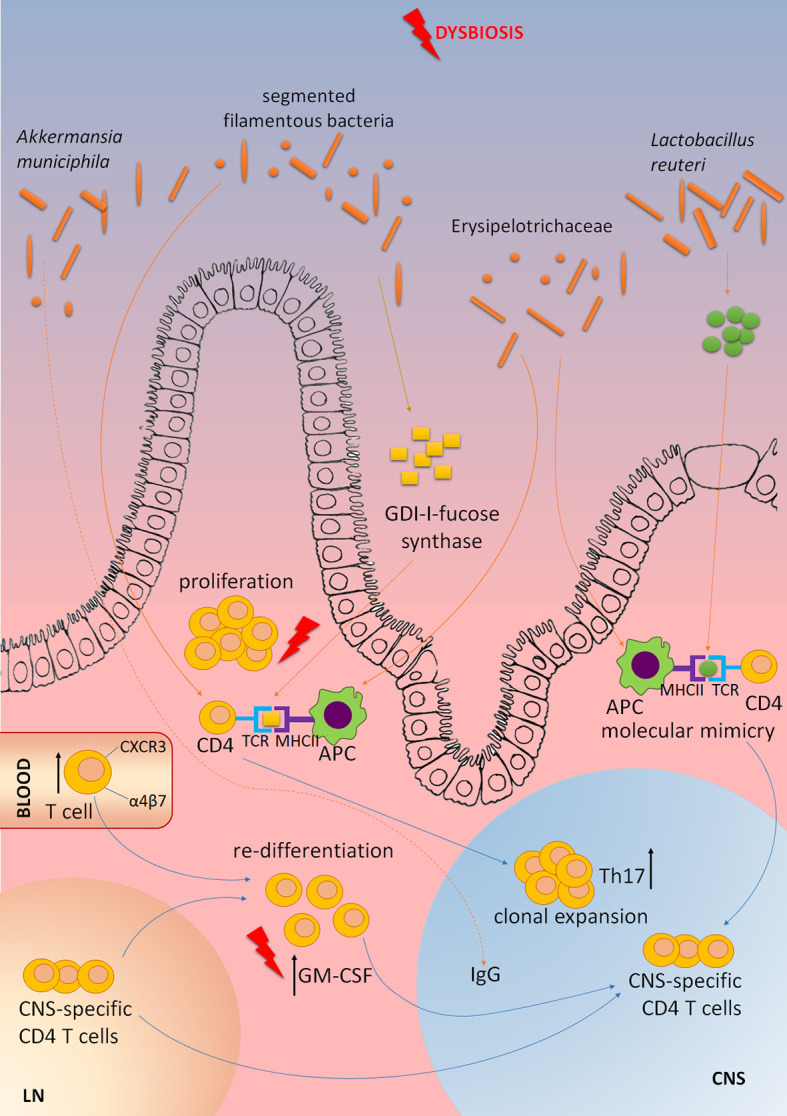
The contribution of gut microbiota dysbiosis to MS pathogenesis. Elevated levels of *Akkermansia muciniphila*-specific IgG are present in the cerebrospinal fluid of MS patients. CD4^+^ T cell clone that is clonally expanded in MS brain lesions is shown to recognize GDP-l-fucose synthase, an enzyme expressed by gut microorganisms. Peptides originating from *Lactobacillus reuteri* mimic myelin oligodendrocyte glycoprotein (MOG), while *Erysipelotrichaceae* can act as an adjuvant to enhance activity of antigen-presenting cells (APC), and subsequent activation of encephalitogenic Th17 cells. Segmented filamentous bacteria stimulate CNS autoimmunity by inducing Th17 cell differentiation. Dysbiosis of gut microbiota in MS patients correlates with increased abundance of CXCR3^+^ T cells expressing the gut-homing α4β7 integrin receptor in the peripheral blood. Gut dysbiosis might increase the abundance of GM-CSF-producing CD4^+^ T cells that are among the major culprits in CNS autoimmunity. CNS-specific T cells originating in lymph nodes (LN) migrate to the gut where they can undergo re-differentiation into potent encephalitogenic cells under the influence of gut microbiota dysbiosis.

As previously emphasized, ILC3 have a central role in controlling the interaction between the gut microbiota and the host immune system. MS patients were shown to have altered gut microbiota composition, and the alterations were associated with MS pathogenesis [reviewed in (84)]. Some studies directly showed the influence of MS gut microbiota on CNS autoimmunity. In a groundbreaking study performed by Wekerle’s group, RR mice that develop spontaneous EAE were transferred with fecal samples obtained from monozygotic twin pairs discordant for MS (85). Germ-free RR mice did not develop EAE, but the disease was initiated through their colonization with human gut microbiota. Importantly, the markedly higher proportion of mice developed EAE in response to MS twin-derived fecal samples than to healthy twin-derived ones. Similar results were obtained in another study, where the transfer of gut microbiota from MS patients to germ-free C57BL/6 mice increased their susceptibility for the induction of active EAE to a greater extent than the transfer of gut microbiota from healthy subjects (86). These studies imply that the dysbiotic gut microbiota of MS patients can be associated with the disease pathogenesis. Indeed, reduced diversity of gut microbiota in MS patients correlated with increased abundance of CXCR3^+^ T cells expressing the gut-homing α4β7 integrin receptor in the peripheral blood (87). Even more, MS gut microbiota might contain microorganisms that are able to provoke or promote CNS autoimmunity. It was reported that elevated levels of *Akkermansia muciniphila*-specific IgG were present in the cerebrospinal fluid of MS patients (88). Moreover, a CD4^+^ T cell clone that was clonally expanded in MS brain lesions was shown to recognize guanosine diphosphate-l-fucose synthase, an enzyme expressed by gut microorganisms (21). Accordingly, a recent EAE study has identified specific gut microorganisms that are involved in the reactivation of MOG-specific T cells (22). Namely, peptides originating from *Lactobacillus reuteri* mimic MOG, while *Erysipelotrichaceae* has been shown to act as an adjuvant to enhance the responses of encephalitogenic Th17 cells. Also, gut microbiota composition was shown to change during EAE and to vary between the disease stages and between different clinical subtypes of the disease (89–91). The contribution of gut dysbiosis to the CNS autoimmunity is shown in **Figure 3**, while the possibility to alter gut microbiota for the benefit of MS patients is discussed in **Box 2**.

Box 2 **Gut microbiota alteration for MS therapy**

Modulation of the gut microbiota that was shown effective in EAE, and investigated in MS trials can be achieved by the application of antibiotics, probiotics, and gut microbiota transfer. Gut microbiota composition modulation by broad-spectrum antibiotics before EAE induction reduced the clinical severity of the disease (92–94), while the therapeutic application was inefficient (95). Still, EAE aggravation as the consequence of broad antibiotic application was observed in rats (89). Minocycline has been considered as a potential therapeutic for MS (96), and its effectiveness in the prevention of clinically isolated syndrome transition into definitive MS was evaluated in a clinical study (97).

Various probiotics were shown safe and efficient in the prophylactic or therapeutic treatment of EAE (6, 98–101). Effects of probiotics were associated with reduced Th1/Th17 presence and activity in lymph nodes draining the site of immunization, in the spleen, and in the blood (100, 101). Probiotics are widely used in humans and are generally safe for prolonged use. However, their ability to modulate the composition of already established gut microbiota or even to re-establish well-balanced gut microbiota after antibiotic-induced depletion is uncertain (102, 103). Maybe the ingestion of prebiotics, i.e. dietary fibers, that help homeostatic bacteria to overwhelm pro-inflammatory ones is a better approach for the treatment of MS. Indeed, there is an ongoing clinical trial: “Prebiotic vs Probiotic in Multiple Sclerosis“ (NCT04038541) that is exploring this possibility. Dietary fibers are metabolized by gut bacteria to short-chain fatty acids (SCFA) that were shown to support gut ILC (51).

The efficiency of fecal microbiota transfer (FMT) has been demonstrated in EAE (104, 105). Some preliminary studies of FMT in a limited number of subjects suggest that this approach can be beneficial in MS (106, 107). Although the results of the studies are encouraging, additional data obtained from large cohorts of patients are needed to get insight into the safety and efficiency of FMT for the treatment of MS. Currently, there are two ongoing clinical trials on the application of FMT in MS (“Fecal Microbiota Transplantation (FMT) of FMP30 in Relapsing-Remitting Multiple Sclerosis (MS-BIOME)”, NCT03594487; “Safety and Efficacy of Fecal Microbiota Transplantation”, NCT04014413).

Numerous data obtained in EAE imply that gut microbiota modulation by antibiotics, probiotics, and by gut microbiota transfer is the feasible way for the prevention and treatment of CNS autoimmunity (108). Still, it has been postulated that appropriate gut immune system development is established under the influence of gut microbiota in the process of “weaning reaction” during the short window of opportunity period, i.e. days 14 to 28 postpartum in mice (109). This reaction is presumably essential for the development of Treg in the gut and prevention of the future inflammatory pathologies in adult organisms. Also, it has been shown that adult gut microbiota composition changes induced by antibiotics and probiotics are not long-lasting, as the gut microbiota tends to get back in the status of the equilibrium with the host genetics (102, 103, 110). Thus, it is reasonable to question if the gut microbiota-directed intervention in adults will be effective in counteracting gut-related inflammatory and autoimmune disorders.

Thus, it is tempting to speculate that gut dysbiosis observed in MS affects ILC3, as these cells are among the central knots of the gut-CNS MS-related network. Accordingly, it seems reasonable to potentiate the regulatory properties of intestinal ILC3 through modulation of gut microbiota for the benefit of MS patients. Modulation of gut microbiota by antibiotics, pro/prebiotics or fecal microbiota transfer (FMT) is one of the ways to influence ILC3, among the other gut immune cells that are responsive to the changes in the gut microbiota composition and function.

Gut ILC3 gene expression profile was shown rather resistant to broad-spectrum antibiotics, unlike ILC1 and ILC2 which had profound changes in the transcriptome (111). Moreover, ILC1 and ILC2 transcriptional profiles were more similar to ILC3 transcriptional profile, under the influence of antibiotics. It will be important to determine if minocycline or some other antibiotic of choice for the treatment of MS, influences regulatory gut ILC3 properties in EAE or other models of MS. Also, dietary fibers could be investigated in conjunction with ILC3 regulatory activity in MS. Yet, it is even more appealing to administer SCFA or agonists of their receptors to potentiate ILC3-mediated CNS autoimmunity amelioration, as discussed in detail below. The effect of FMT on gut ILC3 has not been investigated in MS animal models, and it surely deserves attention.

Specific targeting of gut ILC3 for the benefit of MS patients can be attempted through the application of compounds that influence ILC3 directly or indirectly. Among various compounds that can be used to target gut ILC3, polysaccharide A, AhR agonists, and SCFA are discussed here. Capsular polysaccharide A produced by *Bacteroides fragilis* was extensively studied in the context of CNS autoimmunity. The studies revealed that polysaccharide A acted through TLR2 to stimulate Treg, either directly or by the potentiation of tolerogenic DC functions (112, 113). TLR2 is expressed on gut ILC3 (114) and it will be important to determine if polysaccharide A potentiates regulatory effects of gut ILC3 in EAE.

ILC3 can sense diet-based compounds and changes in the gut microbiota through AhR (115). AhR is highly expressed in ILC3 and is essential for the maintenance of their phenotype under inflammatory conditions (116). For instance, kynurenine produced in gut epithelial cells was shown to increase the abundance of IL-22-producing ILC3 (117). The circulating levels of AhR agonists in general and tryptophan metabolites, in particular, are decreased in sera of MS patients (118). Several research papers indicate the beneficial effects of various AhR ligands in the treatment of EAE (118–120). Notably, EAE enhanced by antibiotics-imposed gut microbiota dysbiosis in mice was ameliorated by AhR ligands indole, indoxyl-3-sulfate, indole-3-propionic acid and indole-3-aldehyde, or the bacterial enzyme tryptophanase (118). Thus, the effects of AhR-based interventions on gut ILC3 functional properties in EAE deserve particular attention.

ILC3 express various SCFA receptors, but the highest expression was shown for free fatty acid receptor 2 (FFAR2 or GPR43), while the expression of FFAR3 (GPR41) was much lower. Also, ILC3 have a higher expression of FFAR2 than other ILC populations (51, 111). SCFA are important for ILC3 homeostasis in the gut, as it was demonstrated that dietary fibers metabolized by gut microbiota to SCFA stimulated ILC3 proliferation in the small intestine *via* upregulating mTOR activity (51, 121). Fecal SCFA levels are decreased in EAE (122), as well as in MS patients (123–125). Accordingly, oral application of dietary fibers or SCFA was shown beneficial in EAE, as they promoted Treg and ameliorated the disease (126, 127). Interestingly, effects of propionate were superior to those of acetate and butyrate (127), and it was supplementation of propionic acid to multiple sclerosis patients that led to Treg/Th17 balance shift towards the regulatory arm and the improvement of the disease course (124). The effect on the disease included reduced annual relapse rate, stabilization of the disability, and decreased brain atrophy after three years of propionic acid intake (124). It has been suggested that acetate and propionate stimulate, while butyrate inhibits innate immune cell activity (128). As FFAR2, in contrast to FFAR3, has a higher affinity for binding acetate and propionate than butyrate (128), it is reasonable to assume that specific activation of FFAR2 is the proper way to stimulate ILC3. Indeed, the deficiency of FFAR2 in ILC3 led to a decrease in their homeostatic proliferation and IL-22 production (41). Further, acetate was shown to promote IL-1β-imposed ILC3 production of IL-22 as a part of its beneficial effects in *Clostridium difficile* infection (71), while butyrate reduced abundance of NKp46^+^ ILC3 in terminal ileal Peyer’s patches, decreased GM-CSF expression in ILC3 and consequently reduced Treg and enhanced antigen-specific T-cell proliferation (129). Moreover, increased fecal butyrate levels correlated with EAE aggravation in antibiotic-treated rats (90). Therefore, it seems rational to insist on the application of the selective FFAR2 agonists, such as the one used in the study of Chun and colleagues. This selective FFAR2 agonist acted preferentially on gut ILC3, increasing their abundance and their IL-22 production (41). Thus, investigation of ILC3-mediated effects of the FFAR2 agonist in EAE is warranted.

**Figure 4:**
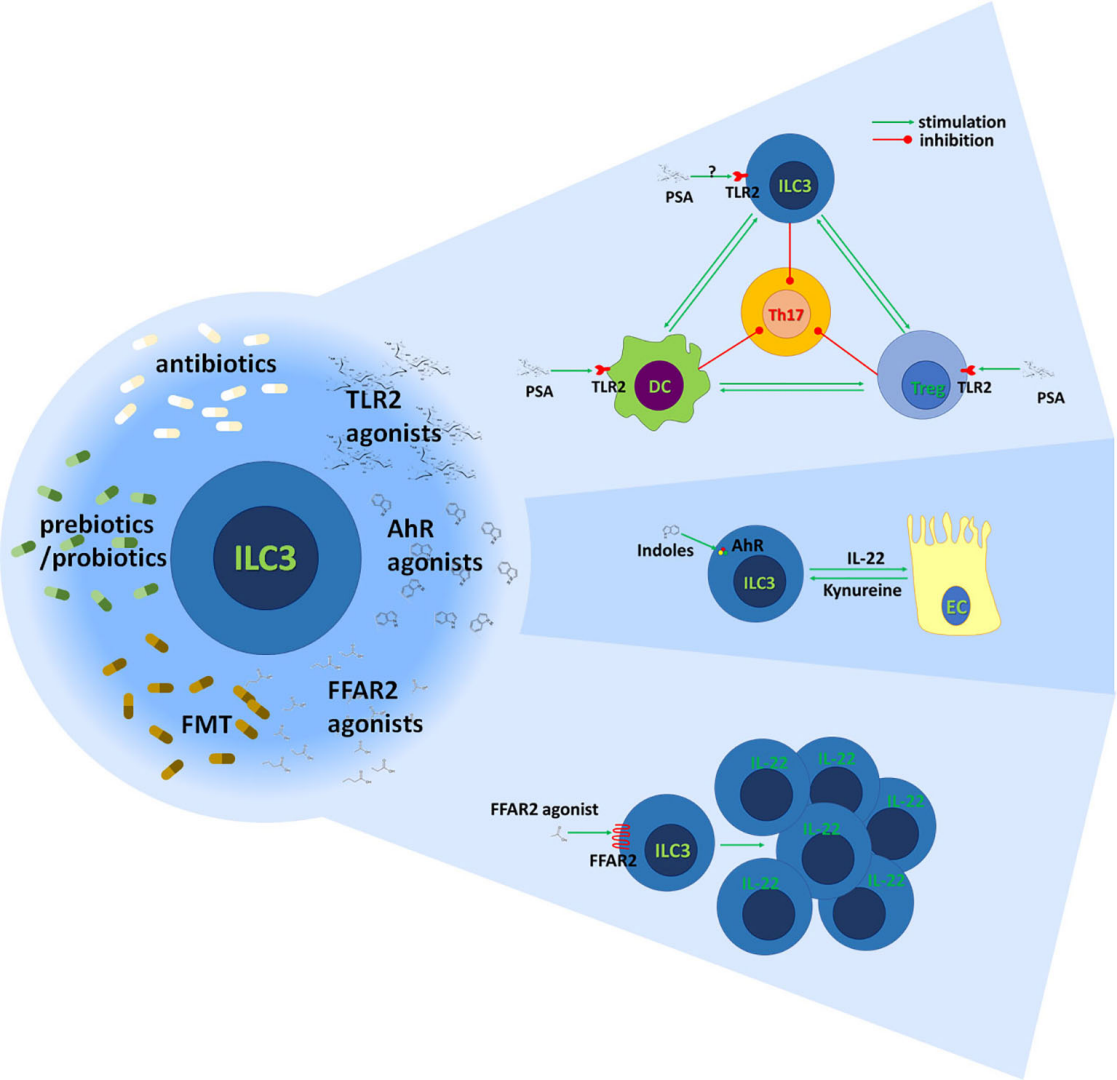
Stimulation of gut ILC3 for the benefit of MS patients. The immunoregulatory activity of gut ILC3 could be achieved through modulation of gut microbiota by antibiotics, prebiotics or probiotics, and fecal microbiota transfer (FMT). Also, it can be potentiated through agonists of TLR2, AhR, and FFAR2. It is known that polysaccharide A (PSA) acts through TLR2 on Treg and DC to inhibit encephalitogenic Th17 cells. Also, it is established that ILC3 potentiate tolerogenic DC properties and stimulate Treg functions. It remains to be determined if PSA acts on ILC3 through TLR2 and if it contributes to ILC3-imposed immunoregulation in the gut. Indole derivates of food and gut microbiota and kynurenine produced by gut epithelial cells (EC) stimulate ILC3 through AhR to generate IL-22. IL-22 has multiple beneficial effects on epithelial cells and the intestinal barrier. FFAR2 agonists promote the proliferation of IL-22-producing ILC3 acting through FFAR2.

Proposed therapeutic interventions for the stimulation of ILC3 immunoregulatory activity are outlined in **Figure 4**.

To conclude, a plethora of data indicates that ILC3 have a central role in gut immune homeostasis, which seems to be essential for the prevention of MS etiopathogenesis. Further, as ILC3 express FFAR2 receptor almost exclusively, they can be easily modulated with respective agonists without affecting other immune cells. Thus, the application of FFAR2 agonists is an excellent therapeutic opportunity. A thorough investigation of the role of ILC3 in the pathogenesis of MS, as well as of the possibility to apply ILC3-directed therapy for the benefit of MS patients is a necessity.

## Author Contributions

All authors drafted the manuscript and participated in the concept design. All authors contributed to the article and approved the submitted version.

## Funding

This work was supported by the Ministry of Education, Science and Technological Development, the Republic of Serbia, contract No. 451-03-9/2021-14/200007.

## Conflict of Interest

The authors declare that the research was conducted in the absence of any commercial or financial relationships that could be construed as a potential conflict of interest.
