# Supplementary material for: ILC3, a Central Innate Immune Component of the Gut-Brain Axis in Multiple Sclerosis
Source: Front Immunol. 2021 Apr 12;12:657622. doi: 10.3389/fimmu.2021.657622 (PMC8071931; doi:10.3389/fimmu.2021.657622)
Supplement: Supplementary file 1 [file Table_1.docx]

Box 1 Multiple sclerosis (MS) essentials

- MS is chronic inflammatory, demyelinating and neurodegenerative disease of the central nervous system (CNS).
- Typical neurological symptoms of MS comprise diminished sensory and visual perception, motor dysfunction, fatigue, pain, and occasionally cognitive deficit.
- Most MS patients exhibit a relapsing-remitting course of the disease, distinguished by alternations between acute attacks and remission phases. Also, MS may present clinically isolated syndrome or progressive (primary and secondary) clinical course.
- Autoimmune response against the CNS resulting in the CNS inflammatory infiltrates has a major contribution to MS pathogenesis.
- IFN-γ-producing Th1 cells and IL-17-producing Th17 cells, defined by the expression of T-bet and RORγt master regulators, respectively, enter the brain at semipermeable and damaged sites of the BBB and initiate neuroinflammation.
- Neuroinflammation induces the opening of BBB and enables the second wave of immune cell entry into the CNS and the formation of brain lesions. CD8^+^ T cells, B cells, and macrophages (Mf) have the leading role in the CNS tissue destruction.
- CD4^+^ T regulatory cells (Treg), defined by the expression of CD25 and Foxp3 as a master transcription factor, operate at the opposite arm of neuroinflammation to reduce/recover damage.
- The etiology of MS is multifactorial and involves interaction between intrinsic (genetic) and extrinsic (environmental) risk factors that influence either innate or adaptive immunity.
- Despite the conclusive autoimmune trait of MS, the precise trigger for the CNS-directed autoimmune response is still unknown.
- Autoreactive T cells in the blood of MS patients display specificity for multiple myelin protein-derived antigens such as myelin basic protein (MBP), proteolipid protein, and myelin oligodendrocyte glycoprotein (MOG). However, none of these myelin protein-derived antigens is recognized as a dominant antigen in MS, while T cells of the same specificity exist in the blood of healthy individuals.
- Experimental autoimmune encephalomyelitis (EAE) is an animal model of MS that is induced in susceptible animals through immunization with the CNS antigens.
