# Supplementary material for: ILC3, a Central Innate Immune Component of the Gut-Brain Axis in Multiple Sclerosis
Source: Front Immunol. 2021 Apr 12;12:657622. doi: 10.3389/fimmu.2021.657622 (PMC8071931; doi:10.3389/fimmu.2021.657622)
Supplement: Supplementary file 2 [file Table_2.docx]

Box 2 Gut microbiota alteration for MS therapy

Modulation of the gut microbiota that was shown effective in EAE, and investigated in MS trials can be achieved by the application of antibiotics, probiotics, and gut microbiota transfer. Gut microbiota composition modulation by broad-spectrum antibiotics before EAE induction reduced the clinical severity of the disease (Ochoa-Repáraz et al., 2009; Miller et al., 2015; Seifert et al., 2018), while the therapeutic application was inefficient (Gödel et al., 2020). Still, EAE aggravation as the consequence of broad antibiotic application was observed in rats (Stanisavljević et al., 2019). Minocycline has been considered as a potential therapeutic for MS (Chauhan et al., 2020), and its effectiveness in the prevention of clinically isolated syndrome transition into definitive MS was evaluated in a clinical study (Metz et al., 2017).

Various probiotics were shown safe and efficient in the prophylactic or therapeutic treatment of EAE (Maassen and Claassen, 2008; Lavasani et al., 2010; Kwon et al., 2013; Yamashita et al., 2017; He et al., 2019). Effects of probiotics were associated with reduced Th1/Th17 presence and activity in lymph nodes draining the site of immunization, in the spleen, and in the blood (Yamashita et al., 2017; He et al., 2019). Probiotics are widely used in humans and are generally safe for prolonged use. However, their ability to modulate the composition of already established gut microbiota or even to re-establish well-balanced gut microbiota after antibiotic-induced depletion is uncertain (Suez et al., 2018; Zmora et al., 2018). Maybe the ingestion of prebiotics, *i.e.* dietary fibers, that help homeostatic bacteria to overwhelm pro-inflammatory ones is a better approach for the treatment of MS. Indeed, there is an ongoing clinical trial: “Prebiotic vs Probiotic in Multiple Sclerosis“ (NCT04038541) that is exploring this possibility. Dietary fibers are metabolized by gut bacteria to short-chain fatty acids (SCFA) that were shown to support gut ILC (Sepahi et al., 2020).

The efficiency of fecal microbiota transfer (FMT) has been demonstrated in EAE (Stanisavljević et al., 2018; Li et al., 2020). Some preliminary studies of FMT in a limited number of subjects suggest that this approach can be beneficial in MS (Makkawi et al., 2018; Engen et al., 2020). Although the results of the studies are encouraging, additional data obtained from large cohorts of patients are needed to get insight into the safety and efficiency of FMT for the treatment of MS. Currently, there are two ongoing clinical trials on the application of FMT in MS (“Fecal Microbiota Transplantation (FMT) of FMP30 in Relapsing-Remitting Multiple Sclerosis (MS-BIOME)”, NCT03594487; “Safety and Efficacy of Fecal Microbiota Transplantation”, NCT04014413).

Numerous data obtained in EAE imply that gut microbiota modulation by antibiotics, probiotics, and by gut microbiota transfer is the feasible way for the prevention and treatment of CNS autoimmunity (Kohl et al., 2020). Still, it has been postulated that appropriate gut immune system development is established under the influence of gut microbiota in the process of “weaning reaction” during the short window of opportunity period, *i.e.* days 14 to 28 postpartum in mice (Al Nabhani et al., 2019). This reaction is presumably essential for the development of Treg in the gut and prevention of the future inflammatory pathologies in adult organisms. Also, it has been shown that adult gut microbiota composition changes induced by antibiotics and probiotics are not long-lasting, as the gut microbiota tends to get back in the status of the equilibrium with the host genetics (Suez et al., 2018; Zmora et al., 2018; Ng et al., 2019). Thus, it is reasonable to question if the gut microbiota-directed intervention in adults will be effective in counteracting gut-related inflammatory and autoimmune disorders.
